# The Added Value of Musculoskeletal Simulation for the Study of Physical Performance in Military Tasks

**DOI:** 10.3390/s21165588

**Published:** 2021-08-19

**Authors:** Ilona Kessels, Bart Koopman, Nico Verdonschot, Marco Marra, Kaj Gijsbertse

**Affiliations:** 1 Orthopaedic Research Laboratory, Radboud Institute for Health Sciences, Radboud University Medical Center, 9101, 6500 HB Nijmegen, The Netherlands; nico.verdonschot@radboudumc.nl; 2 Laboratory of Biomechanical Engineering, Faculty of Engineering Technology, University of Twente, 217, 7500 AE Enschede, The Netherlands; h.f.j.m.koopman@utwente.nl (B.K.); marco.marra@radboudumc.nl (M.M.); 3 Training and Performance Innovations, TNO, 23, 3769 ZG Soesterberg, The Netherlands; kaj.gijsbertse@tno.nl

**Keywords:** inertial sensors, Xsens, musculoskeletal modeling, AnyBody, military, kinematics, energy expenditure, muscle parameters

## Abstract

The performance of military tasks is often exacerbated by additional load carriage, leading to increased physical demand. Previous studies showed that load carriage may lead to increased risk of developing musculoskeletal injuries, a reduction in task speed and mobility, and overall performance degradation. However, these studies were limited to a non-ambulatory setting, and the underlying causes of performance degradation remain unclear. To obtain insights into the underlying mechanisms of reduced physical performance during load-carrying military activities, this study proposes a combination of IMUs and musculoskeletal modeling. Motion data of military subjects was captured using an Xsens suit during the performance of an agility run under three different load-carrying conditions (no load, 16 kg, and 31 kg). The physical performance of one subject was assessed by means of inertial motion-capture driven musculoskeletal analysis. Our results showed that increased load carriage led to an increase in metabolic power and energy, changes in muscle parameters, a significant increase in completion time and heart rate, and changes in kinematic parameters. Despite the exploratory nature of this study, the proposed approach seems promising to obtain insight into the underlying mechanisms that result in performance degradation during load-carrying military activities.

## 1. Introduction

Soldiers are often involved in various physically demanding tasks, performed under adverse circumstances during military operations. Load carriage is an important component thereof. During training or mission deployment, soldiers wear bulky and heavy personal protective clothing (PPC) (e.g., body armor and helmet), combat specific and personal protective equipment (PPE) (e.g., weapon systems and communication devices), and provisions. Over the years, the weight of these loads has only increased by the rapidly available new technologies that aim to enhance protection, firepower, and communication of soldiers [1]. These additional external loads complicate these operations even more and may lead to serious consequences. Examples are increased energy expenditure, fatigue, and obstruction of physical performance, but also the inability to perform a task. Heavy loads reduce mobility and speed [2], making the soldiers more susceptible to enemy threats [3,4]. Additionally, exposure to these heavy loads often lead to overload and musculoskeletal injuries among soldiers, causing reduced work capacity and combat readiness [5].

To better understand the impact on the soldier’s physical performance by typical military loads, the most research has focused on measuring obstacle course completion time for various loading conditions [2,6,7,8,9] and generally, in the context of body worn load, the reported reduction in physical performance is approximately 1.5% for every kilogram of carried mass [10]. However, these empirical methods do not provide any insight into the underlying mechanisms of performance degradation. The analysis of kinematic parameters, joint loading, and metabolic costs are important to comprehend the full effect of carried load on military performance. Examples of altered body movements during load are an increase in trunk movement in the sagittal plane [11,12,13,14,15,16,17] associated with an increase in the craniovertebral angle [13] and a decrease in the position of the center of mass (CoM) [11]. Next to this, physical loading was found to change the range of motion of the ankle, knee, and hip angle [11,12,13,17,18,19,20]. In addition to the kinematic consequences, increases in knee, hip, and ankle moments were found [11,17,19,20], but also increases in ground and joint reaction forces [8,14,20], muscle activities, and muscle burdening [11,13,16,20].

Other physiological loading metrics also increase, such as heart rate [19,21,22,23] or energy expenditure (i.e., metabolic costs) [1,8,14,19,20,23,24] that are associated with increased external loads. The reliable estimation of energy expenditure is important, since predictions or guidelines rely on this parameter. For example, body core temperature and sweat rating are important parameters that are used to prescribe work–rest regimes to prevent heat strokes and these calculations rely on energy expenditure of the undertaken activities [25]. Additionally, energy expenditure is used to calculate the maximum acceptable work duration [26], which can be used for planning of load carriage tasks and/or managing personnel undertaking these tasks. For example, it would be valuable to know how much energy a series of tasks during a mission would cost in order to manage personnel, pacing, or nutrition strategies.

Unfortunately, the available tools to assess these performance metrics are crude or require a specific laboratory setting, i.e., optical motion-capture systems, that are impractical for military activities. To overcome these shortcomings, we propose a combination of an inertial measurement unit (IMU) system and musculoskeletal modeling as a convenient and ambulatory means to study physical performance in military activities. Musculoskeletal models have already proven their added value with regard to human movement research in several disciplines, such as sports, ergonomics, product design in industry, and for clinical purposes [27,28,29,30]. To our knowledge, only Lenton et al. [31,32,33] used musculoskeletal modeling to determine the effects of load carriage on military performance. However, their study was limited to the use of an optical motion-capture system and force plates, which are impractical for field evaluations.

The primary goal of this explorative study is to determine whether the combination of an IMU system and musculoskeletal modeling can provide additional insights into the underlying mechanisms of performance degradation indued by physical loading in military activities. This can help in determining the limitations of physical performance and to identify useful relationships between several aspects of load carriage, which are of interest to, e.g., mission planning, development of training regimes, and personnel management.

We anticipated that the model would provide detailed and quantitative data on the underlying mechanisms of physical performance during simulated load-carrying tasks. Our working hypothesis was that increased load carriage leads to altered body-level and muscle-level physical performance during military activities. Specifically, we hypothesized that increased load leads to movement alterations, including a speed decrease, and to changes in muscle force levels, resulting in increased energy expenditure.

## 2. Materials and Methods

### 2.1. Subjects

Data of ten subjects were collected during an experiment (age = 24.2 ± 2.7 years, body weight = 93.2 ± 8.5 kg, body height = 188.9 ± 5.9 cm). All subjects had an operational function as military at the moment of the experiment. The inclusion criteria were: male gender, age 18–30 years, no reported physical or mental health issues, and experience with military training (specifically, obstacle course) or deployment. Subjects were informed that their participation was voluntary, and they could quit the experiment at any time. At the start of the experiment, subjects gave written consent. The study was approved by the internal ethical review board of TNO and registered under number 2019-094.

### 2.2. Measurement Setup

The experiment consisted of the performance of an agility run with different loading conditions. The agility run is an obstacle of the military obstacle course as developed in the Load Effects Assessment Program (LEAP) (SOLIID-LEAP, Soesterberg, The Netherlands). The LEAP obstacle course is an instrumented operationally realistic combat mobility course and comprises ten sequential timed mobility tasks. These tasks were designed to simulate the most common or most challenging physical tasks encountered during military tactical operations and are used to assess operationally relevant mobility and physical performance [34,35].

**Figure 1:**
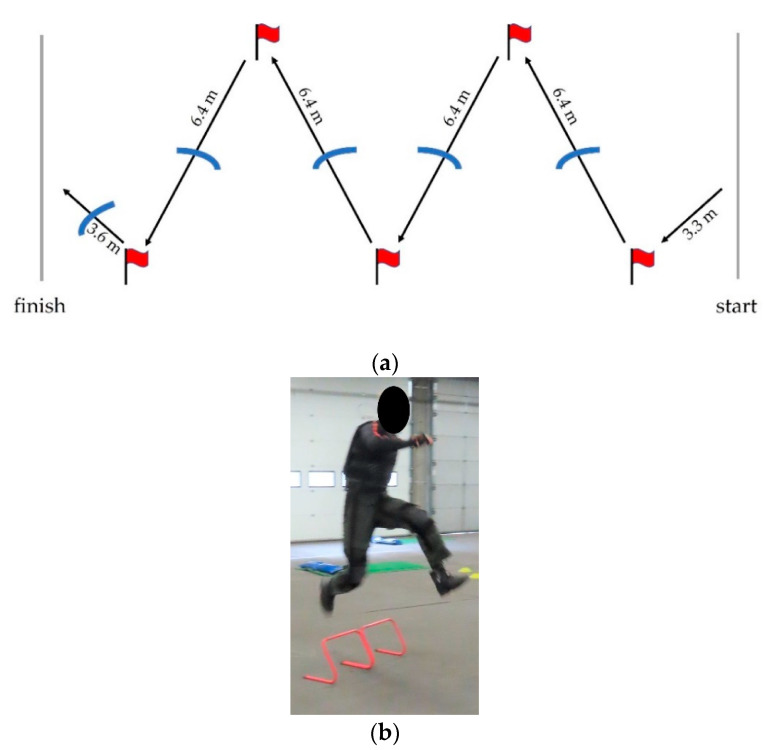
(**a**) Overview of the agility run with five flags and five (low) hurdles. The starting line is on the right side of the image and the finishing line on the left side. (**b**) Subject is jumping over one of the hurdle obstacles.

The agility run represents a run through a complex terrain. The set-up consists of a 32.5 m zig-zag course with five flags and five (low) hurdles, demonstrated in Figure 1a. The subjects were instructed to use maximum speed to go from the start to the finish following the course of the flags, while jumping over the hurdle obstacles (Figure 1b). To maintain consistency in the performance, subjects needed to choose a strategy to perform the agility run: they were instructed to start running with the same leg all tests, to jump over the obstacles the same way all tests, and to make the cut around the flags the same way all tests. Each participant practiced their strategy five times while wearing 15.6 kg of extra load, which was considered to be enough to diminish a learning effect during the experiment [36].

**Figure 2:**
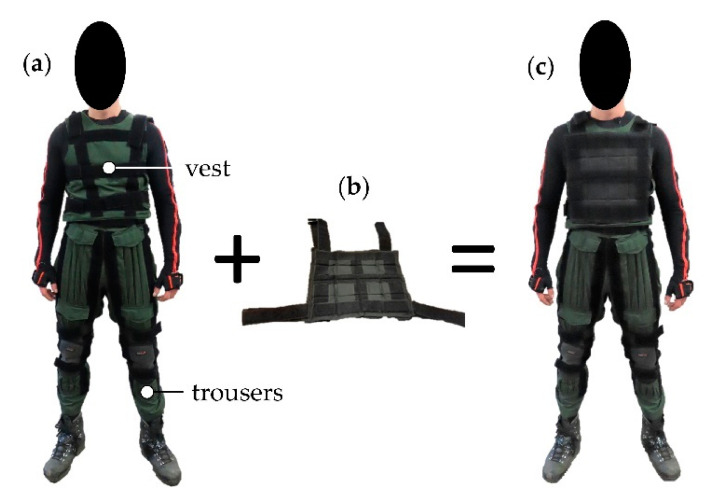
The agility suit worn during the experiment. (**a**) Subject is wearing the vest and pair of trousers. (**b**) High density mass pads of 5.2 kg each can be added to investigate the effect of weight. (**c**) The mass pads are attached to the vest. The load is divided on the front and back of the subject (details are given in Table 1).

To realize different loading conditions, a custom build agility suit was used. This suit enables alterations of the individual effects of PPC/E weight, bulk, and stiffness. In this experiment, only the effect of weight was investigated, which requires the use of the vest to add high density mass pads of 5.2 kg each. The mass pads were attached to the chest and back, resembling a tactical vest or double pack, a common way of load carriage in military [1]. Additionally, the trousers of the agility suit were worn to protect the participant and equipment, but no mass was attached there. The agility suit is shown in Figure 2.

During the experiment, completion time of the agility run was registered with a handheld stopwatch. Additionally, heart rate was measured with a Polar H10 sensor chest strap (Polar Electro, Kempele, Finland) during the agility run and a rest period of two minutes afterwards. Full-body kinematics were recorded with an Xsens suit consisting of 17 inertial measurements units (IMUs) (Xsens MVN Link, Xsens Technologies B.V., Enschede, The Netherlands). With use of the affiliated software, MVN Studio 2019.2.1, the human movement during the performance of the agility run was recorded in real-time with a sample frequency of 240 Hz. Anthropometric data were measured with a segmometer and entered into the software. These data are used by the software to scale the body segments based on an anthropometric model to obtain a subject-specific model. Additionally, body weight was measured. Prior to the measurements, sensor calibration was performed according to the instructions of Xsens (N-pose and walk protocol).

Motion capture data were used as input for the musculoskeletal model (see Section 2.4). Prior to using the data as input, the Xsens data were HD reprocessed in MVN Studio. Thereafter, the files were manually synchronized by finding the start and end of the movement in the recordings, to save only the parts of the agility run, and exported as BVH files.

### 2.3. Measurement Protocol

Three different loading conditions were defined:

- no extra mass (M0): boots and sport clothes (~2.27 kg [36]) + Xsens suit + agility suit (both suits together were 3.1 kg) + 0 kg extra mass;
- light extra mass (M1): M0 + 15.6 kg extra mass (5.2 kg on the front, 10.4 kg on the back);
- heavy extra mass (M2): M0 + 31.2 kg extra mass (15.6 kg on the front, 15.6 kg on the back).

Each condition was performed three times, which resulted in a total of nine tests for each subject. The order was randomized to reduce the influence of fatigue on the performance. In between the tests, subjects needed to take two minutes rest on a chair. Prior to the experiment, subjects were instructed to warm up following given instructions [37].

The experiment continued over two days, with five subjects each day. The experiment was set up indoors at the Scheickbarracks (LEAP—SOLIID, Soesterberg, The Netherlands). Before the experiment, subjects recieved extensive instructions about the experiment.

### 2.4. Musculoskeletal Modeling Environment

A musculoskeletal model was developed in the AnyBody Modeling System v. 7.2.3 (AnyBody Technology A/S, Aalborg, Denmark). The BVH_Xsens model of the AnyBody Managed Model Repository (AMMR) v. 2.2.3 was used as base model. This full-body model allows kinematic input in the form of inertial motion-capture data. The model also provides ground-reaction force (GRF) prediction capabilities that allow the analysis of inverse dynamic models based on recorded motion without GRF force measurement [38,39].

#### 2.4.1. Model Adaptations and Settings

**Figure 3:**
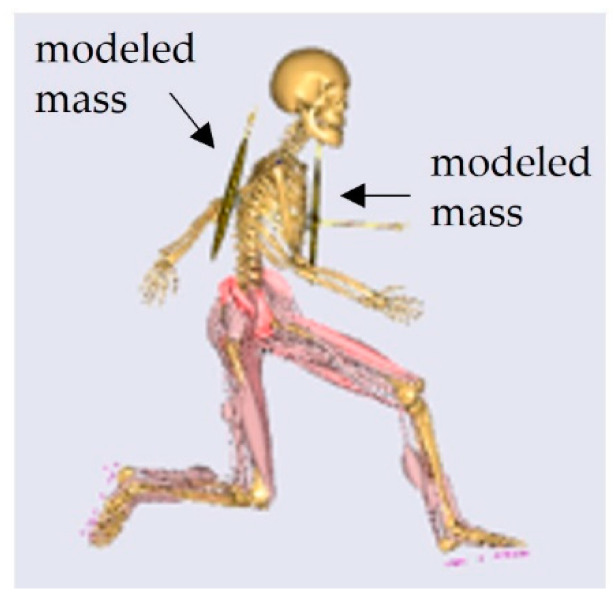
Modeled external load attached to chest and back in the AnyBody modeling system.

The base model was adapted to incorporate the external load. Two additional segments were created representing the worn mass; one rigidly attached to the chest and the other to the back of the thorax segment (Figure 3). These two segments were assigned a mass, equal to the weight of the actual worn masses (Table 1), and the three principal moments of inertia, based on the moment of inertia of a uniform rectangular plate.

#### 2.4.2. Musculoskeletal Simulations

To determine the effect of external loading on energy expenditure and muscle parameters, musculoskeletal simulations were run using the kinematic datasets of each of the three repetitions of both base (M0) and adapted models (M1, M2), resulting in a total of nine simulations. These simulations were only run for one subject due to the complexity of the analysis.

Each simulation consisted of two steps: an inverse kinematic and an inverse dynamic analysis. In the inverse kinematic analysis, virtual marker positions were derived from the Xsens stick-figure model and were used (1) to scale the body segment dimensions and (2) as target for a marker-tracking motion-optimization analysis. The resulting optimized joint kinematics and body segment dimensions were stored for the subsequent inverse dynamic analysis. In this last step, joint kinematics were input to a muscle-actuated inverse dynamic model that solved for muscle forces, and joint- and ground-reaction loads. Additional results of this analysis were the instantaneous metabolic power ($P_{met}$) and the time-integral thereof, i.e., metabolic energy expenditure throughout the agility run ($E_{met}$). For simplicity, only the lower-limb muscles contributed to the metabolic power estimation.

### 2.5. Data Analysis

All data were pseudonymized before analysis. Data analysis was performed with Matlab R2019b (The MathWorks, Inc., Natick, MA, USA). Results on completion time and heart rate were analyzed for all ten subjects. Energy expenditure, muscle parameters, and kinematics parameters were analyzed for only one subject.

#### 2.5.1. Muscle Parameters

The effect of external load on muscle parameters of the gluteus maximus muscle and vastus lateralis muscle was investigated. The parameters for the left and right leg were averaged. Using the simple metabolic energy model [40] and the equation for power, the relation between energy expenditure and these parameters is given by:

(1) $$P_{met}=\eta \;\cdot P_{m}=\eta \cdot F_{m}\cdot {\dot{L}}_{m}$$

where η is an efficiency coefficient, which is 0.25 for concentric contractions and −1.2 for eccentric contractions, $P_{m}$ is mechanical power, $F_{m}$ is muscle force, and ${\dot{L}}_{m}$ is muscle contraction velocity.

#### 2.5.2. Kinematic Parameters

**Figure 4:**
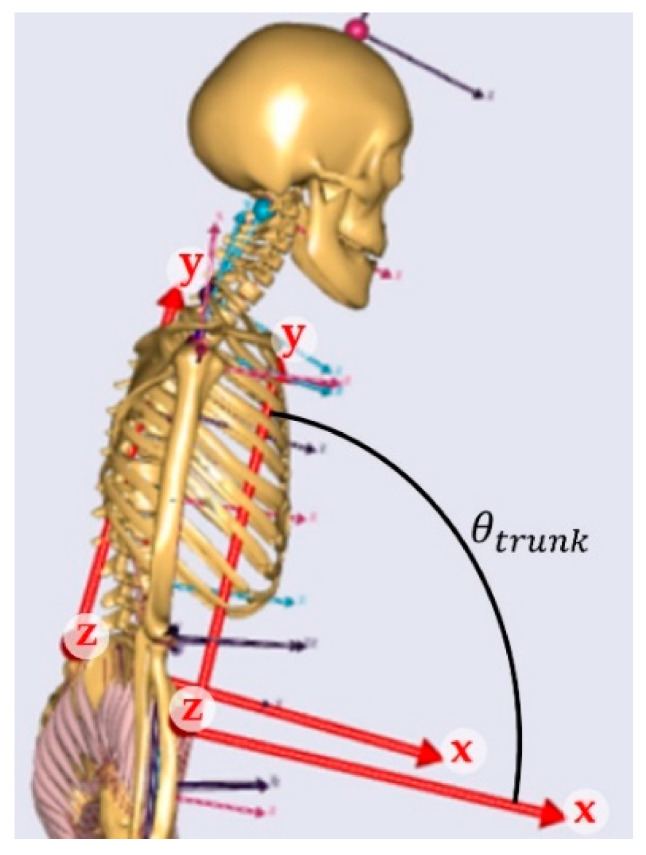
Definition of the trunk angle in the sagittal plane, which is the rotation around the *z*-axis between the anatomical frames of the pelvis and the thorax segments.

Movement alterations as consequence of the added load were investigated for the pelvis:trunk angle in the sagittal plane (flexion/extension) ($\theta_{trunk}$) and the vertical position of the trunk *CoM* ($y_{CoM}$). The trunk angle in the sagittal plane was defined as the rotation around the *z*-axis between the anatomical frames of the pelvis and the thorax segments, which is visualized in Figure 4. The range of motion (*RoM*) of the trunk angle in the sagittal plane and vertical displacement (*VD*) of the *CoM* were calculated:

(2) $$RoM_{trunk}=\max\left(\theta_{trunk}\right)-\min\left(\theta_{trunk}\right)$$

(3) $$VD_{CoM}=\max\left(y_{CoM}\right)-\min\left(y_{CoM}\right)$$

#### 2.5.3. Statistical Analysis

Statistical analysis was performed using IBM SPSS Statistics 26 (IBM Inc., Armonk, NY, USA). A *p*-value equal to or less than 0.05 was considered significant, unless stated differently. Completion time and heart rate data were checked for normality with the Shapiro–Wilk test. Differences between conditions for the several outcome measures were identified with a repeated measures ANOVA test, followed by post hoc tests using Bonferroni correction to account for multiple testing bias. In case of non-normality and for energy expenditure, muscle, and kinematic parameters, differences were identified with a non-parametric Friedman’s ANOVA test for K-related samples, followed by Wilcoxon signed rank test as post hoc test.

## 3. Results

Motion capture data were successfully recorded with the IMU system and reconstructed in the musculoskeletal modeling environment (see Supplementary Video S1). Table 2 presents the results of ten subjects for completion time and heart rate. The results for energy expenditure, muscle and kinematic parameters are shown in Table 3 for one subject.

### 3.1. Completion Time and Heart Rate

**Figure 5:**
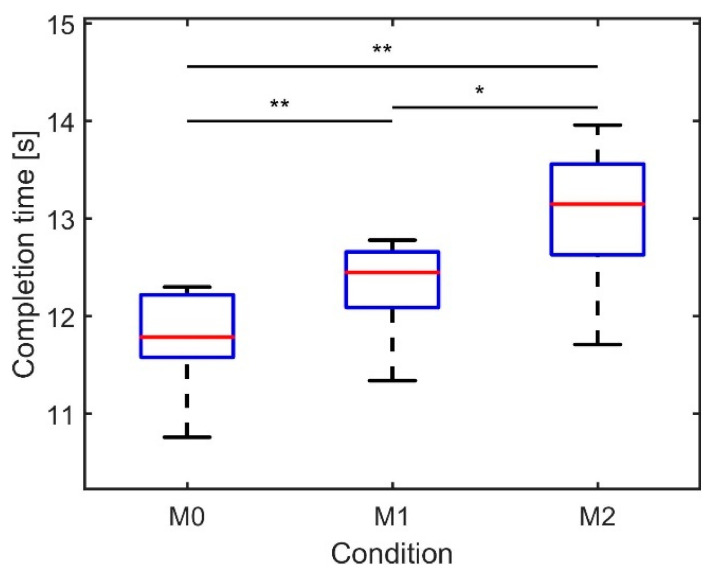
Boxplot of median completion time (s) of the agility run over three repetitions per condition (*n* = 10). The asterisks indicate significant differences between the conditions: * *p* < 0.01, ** *p* < 0.001.

Completion time was found to increase significantly with additional loads (ANOVA, *p* < 0.000). Figure 5 depicts the median completion times over three repetitions per condition for ten subjects, which varied between 11.78 s (M0), 12.32 s (M1), and 13.08 s (M2). Post hoc tests revealed that the differences between all conditions were significant.

**Figure 6:**
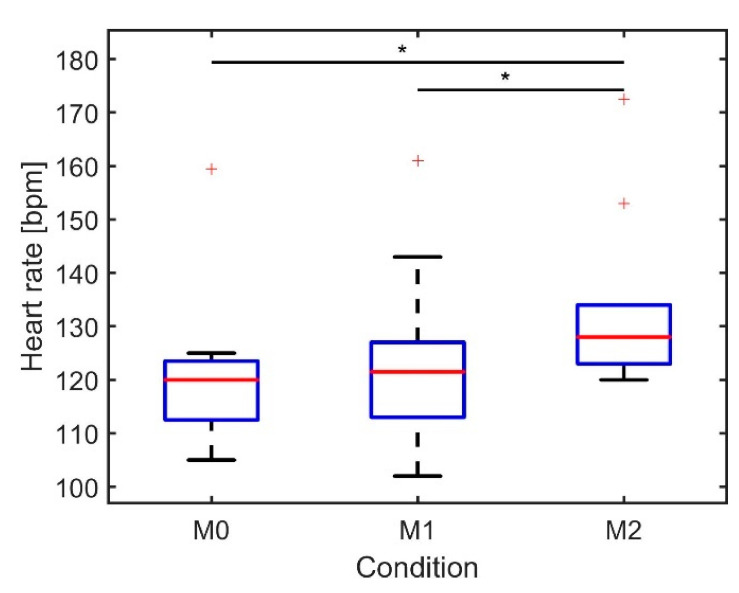
Boxplot of the median heart rate during agility run + 2 min rest afterwards over three repetitions per condition (*n* = 10). The asterisk indicates a significant difference between the conditions: * *p* < 0.01.

Heart rate during the agility run and two minutes rest afterwards was found to be significantly affected by the load (ANOVA, *p* < 0.000) (Figure 6). The post hoc test revealed that median heart rate differed significantly between M0–M2 and M1–M2, with a difference of 8 and 7 bpm, respectively. For all subjects, the highest heart rate values were measured after completion of the agility run.

### 3.2. Energy Expenditure

**Figure 7:**
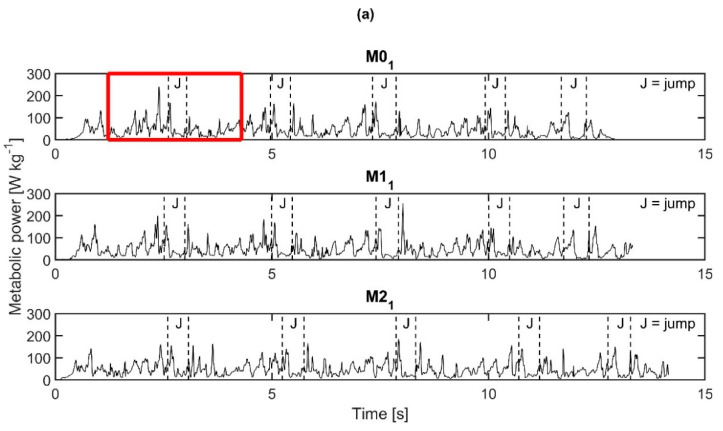

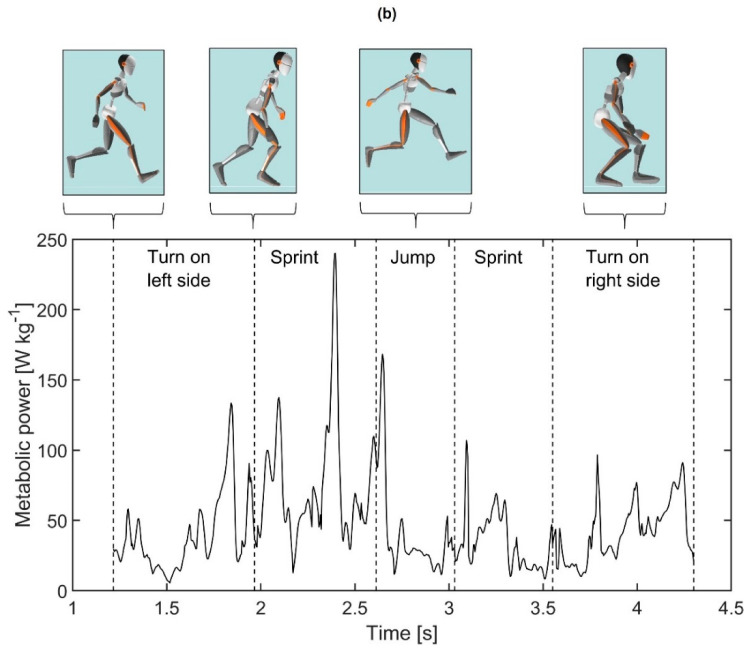
Course of the metabolic power for subject 8. (**a**) Metabolic power normalized to body weight during the agility run. Data from M0_1_, M1_1_, and M2_1_ are shown. The jumps over the hurdles during the run are marked. A part of the agility run of M0_1_, denoted with the red rectangular, is zoomed in on in (**b**). Specific movements during the agility run are marked.

The course of the metabolic power during the agility run for the first repetition of each condition is shown in Figure 7a. Similar patterns in metabolic power were observed between the three conditions, in particular during the marked jumps. These jumps were characterized by a peak in metabolic power just before and at the start of the jump (Figure 7b).

**Figure 8:**
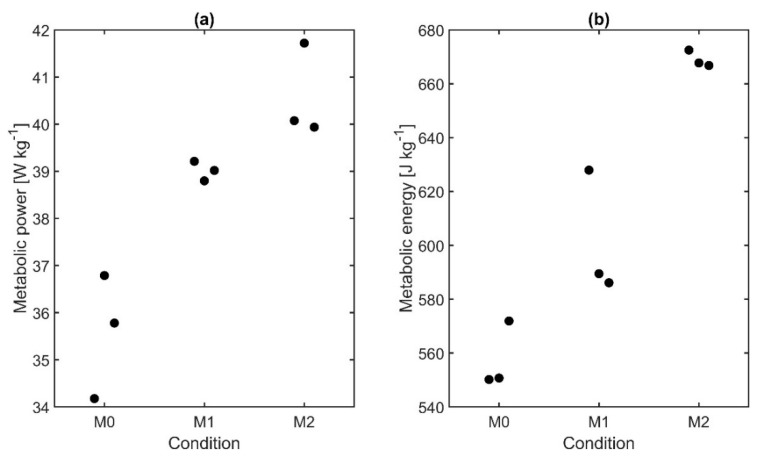
(**a**) Metabolic power (median values) and (**b**) metabolic energy of subject 8 for three repetitions per condition normalized to body weight.

Median metabolic power normalized to body weight varied between 35.8 W kg^−1^ (M0), 39.0 W kg^−1^ (M1), and 40.0 W kg^−1^ (M2) (Figure 8a). Metabolic energy increased from 551 J kg^−1^ for M0 to 589 J kg^−1^ for M1 and 668 J kg^−1^ for M2 (Figure 8b). Both parameters were significantly affected by the load (ANOVA, *p* = 0.050). However, post hoc tests did not reveal any significant difference between the conditions.

### 3.3. Muscle Parameters

**Figure 9:**
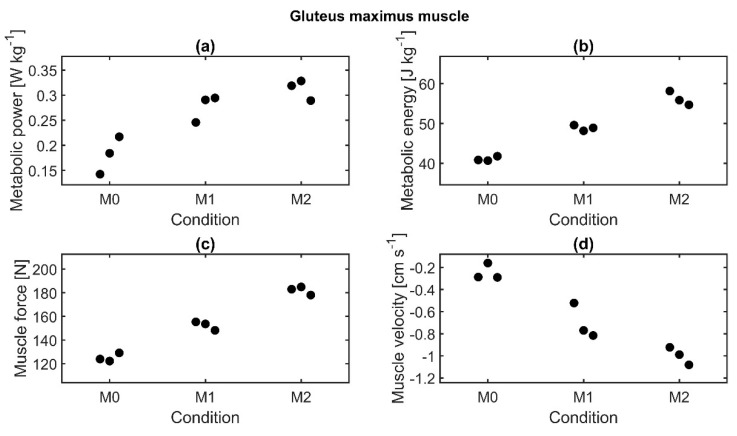
Muscle parameters of the gluteus maximus muscle of subject 8 for three repetitions per condition. Median values are displayed. (**a**) Metabolic power normalized to body weight, (**b**) metabolic energy normalized to body weight, (**c**) muscle force, and (**d**) muscle velocity.

Metabolic power, metabolic energy, and muscle force of the gluteus maximus muscle were found to increase with additional load, while muscle velocity decreased (Figure 9). ANOVA test results showed that metabolic energy, muscle force, and muscle velocity were significantly affected by the load, but post hoc tests did not reveal significant differences between the conditions.

**Figure 10:**
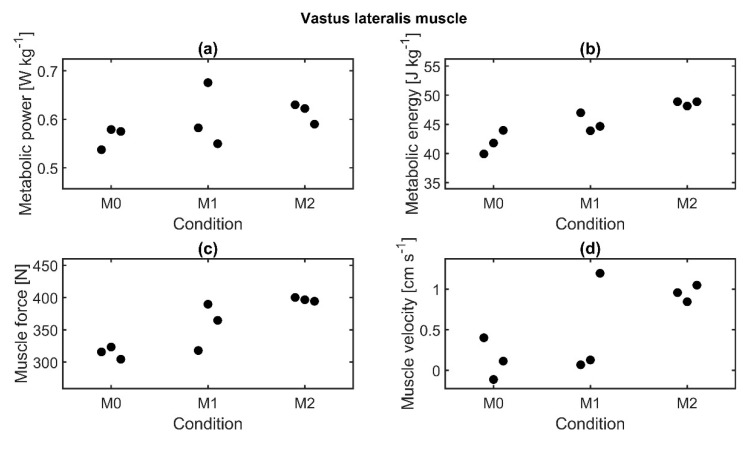
Muscle parameters of the vastus lateralis muscle of subject 8 for three repetitions per condition. Median values are displayed. (**a**) Metabolic power normalized to body weight, (**b**) metabolic energy normalized to body weight, (**c**) muscle force, and (**d**) muscle velocity.

For all four parameters of the vastus lateralis muscle, an increase with additional loads could be seen (Figure 10). Only metabolic energy and muscle force were found to be significantly affected by the load based on ANOVA test results, but again, post hoc tests did not reveal any significant difference between the conditions.

### 3.4. Kinematic Parameters

**Figure 11:**
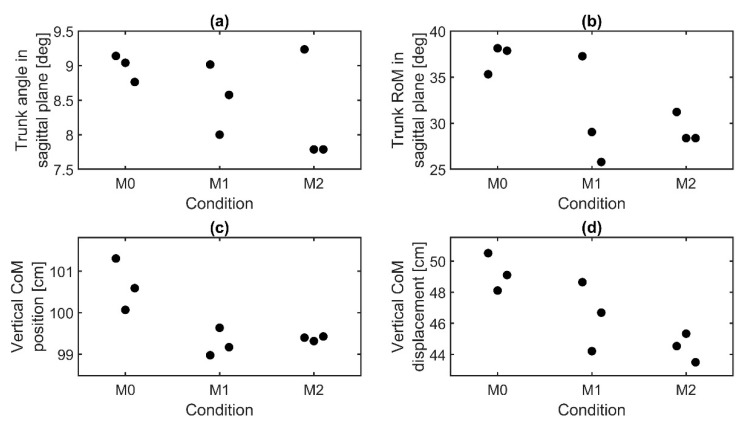
Results of kinematic parameters of subject 8 for three repetitions per condition. Median values are displayed. (**a**) Trunk angle in the sagittal plane, (**b**) trunk range of motion in the sagittal plane, (**c**) vertical position of the center of mass, and (**d**) vertical displacement of the center of mass.

Additionally, the trunk angle and *RoM* in the sagittal plane decreased with additional loads worn, as well as the vertical position and displacement of the *CoM* (Figure 11). However, these parameters were not significantly affected by the load.

## 4. Discussions

The presented method of applying IMUs in combination with musculoskeletal modeling allows for a comprehensive analysis of the effect of carried load on the physical performance during a military obstacle course. With this approach, the functional performance degradation (i.e., completion time) as a result of load carriage can be decomposed in kinematic alterations, together with muscle specific aspects and metabolic costs. The exploratory results of this study illustrate how an increase in carried load leads to changes in movements and, consequently, leads to altered muscle activation and an increase in metabolic costs. Similarly, the completion time of the agility run increased with added load, which is in agreement with our hypothesis.

Energy expenditure did not increase equally between M0–M1 and M1–M2, while load was increased equally. These findings imply that there does not exist a proportional relationship between load carriage and energy expenditure, which was supported by the finding of Looney et al. [41].

Muscle force and muscle contraction velocity are the main muscle parameters that define metabolic power, as indicated by Equation 1. The gluteus maximus muscle and vastus lateralis muscle both showed an increase in muscle force between M0–M1 and M1–M2, while muscle contraction velocity decreased for the gluteus maximus muscle between all conditions and increased for the vastus lateralis muscle. A possible explanation is that more force is required from all muscles to be able to carry the increased load, which consequently requires more energy from the body. To use its energy efficiently, the human body will react to this increased energy demand by changing its movements and muscle activation accordingly. Therefore, muscle contraction velocity may increase for some muscles when load is increasing, while other muscles will decrease their contraction velocity.

The time to complete the obstacle increased significantly with added loads, with an increase of 5.6% between M0–M1 and equally between M1–M2. These results suggest a proportional relationship between load carriage and completion time. Hence, the weight of external load negatively affects functional performance expressed as completion time. These findings were in agreement with our hypothesis and are extensively described in the literature; Vitali et al. [7] showed comparable alterations in completion time. They found a decrease of 0.51% in inverse completion time per kg added load, compared to a 0.36% increase in completion time per kg added load found in this study. Mitchell et al. [36] performed a study with a similar agility run as used in this study and showed higher completion times of 14.75 s on average with a loading condition comparable to our M0 condition, which is 2.99 s slower than our finding. This difference can be explained by the fact that their subjects performed the agility run as part of the full obstacle course, while our subjects only performed the agility run. Therefore, fatigue could be a reason for their longer completion times.

An increase in metabolic power suggests an increase in internal load, which is defined as the relative biological stressors imposed on a human body during physical exertion [42]. One of the objective measures of internal load is heart rate, because it is highly correlated with oxygen consumption during continuous work, which is directly related to energy expenditure [25]. Our results support the hypothesis of increased internal load during load carriage, as significant increases in heart rate were found with added external load. Although several studies showed a linear relationship between load carriage and heart rate [19,22], this conclusion cannot be drawn from our results. An explanation for this could be that oxygen consumption, and with that heart rate, is only directly related to energy expenditure for aerobic activities that require continuous work. However, an agility run is a short, intense activity, which requires the use of the anaerobic pathway. This pathway requires less oxygen consumption and therefore heart rate increases less. Additionally, changes in heart rate are only visible after a certain time with respect to the start of an activity, which was also indicated by the fact that maximum heart rate values were reached after completion of the agility run. Another noteworthy point is that heart rate levels might not have been decreased to resting levels in the short rest periods (two minutes) that we applied in the experimental protocol.

The results of this study show a decrease in trunk angle and RoM in the sagittal plane with added loads. This is in contrast to the results described in the literature [11,13,14,15,16], which showed that, for increased load, subjects tend to lean further forward to counterbalance the effect of added load and maintain stability. However, most of those studies investigated the effect of carrying the load solely on the back, while we distributed the load over the chest and back. Only Park et al. [16] also investigated the effect of load distribution over the chest and back. In agreement with our results, they showed a decrease in trunk movement in the sagittal plane. This probably allowed the subjects to better maintain their stability and reduce vertical oscillations. Next to the decrease in trunk angle, a decrease in vertical position of the CoM was also found in this study. This was in accordance with previous studies [11,14], although they investigated the effect of load carriage using a backpack and explained this by the greater forward lean of the pelvis. Because the subjects in this study had the weight distributed over the chest and back, they probably compensated this extra load by bending more at the knees or hips, while keeping their upper body as straight as possible, again to reduce vertical oscillations.

As mentioned before, we aimed to showcase the possibilities of the presented method with some preliminary results. To that end, we made some decisions and assumptions which we would like to point out. First of all, many studies have investigated the accuracy and reliability of metabolic power estimations using muscle energy models [40,43,44,45,46,47,48,49,50,51]. Four of these models are implemented in the AMMR v. 2.2.3.: the simple mathematic model described by Margaria [40], two models developed by Umberger (2003 [43] and 2010 [44]), and the model developed by Bhargava [45]. The simple model describes the energy conversion from the mechanical domain to the metabolic domain using an efficiency coefficient. Both the Umberger models and the Bhargava model make use of the Hill-type model for muscle contraction [52] and the first law of thermodynamics. Where the Bhargava model is developed based on frog data, the Umberger models are based on human data. Studies that evaluated these models have not yet found enough evidence to favor one of these models over the others [53,54]. Therefore, in this study, we used the simple metabolic energy model.

Secondly, the amount of force a muscle can deliver is dependent on the muscle strength (i.e., maximal isometric force). In this study, muscle strength is scaled uniformly based on body weight. This may not reflect the actual distribution in the military subject. On the other hand, muscle strength is expected to decrease over time during intense activities due to muscle fatigue [55], especially during repeated or sustained muscle contraction [56]. Muscle fatigue is, for instance, related to motor unit activation patterns and muscle fiber contractile properties [56]. Because musculoskeletal fatigue limits are considered one of the aspects of a soldier readiness score, and fatigue limits are often expressed in terms of energy expenditure rates [57], future modeling studies estimating energy expenditure should also incorporate the influence of muscle fatigue.

Thirdly, data on completion time and heart rate were analyzed for ten subjects, and both ANOVA and post hoc test results showed significant changes between the conditions. However, the other data were only analyzed for one subject. Although ANOVA test results showed significant changes due to the increased loading for some of the parameters, the post hoc test results showed no significant changes which may be the result of the limited number of participants in the analysis of metabolic costs.

Future studies should include more participants and focus on validation of the outcome parameters. A previous study demonstrated the use of IMUs in a military context by validating it against a gold standard optical motion capture system [58]. The validity of ground reaction force prediction and musculoskeletal model-based inverse dynamic analysis using IMUs were previously validated for walking activities by Karatsidis et al. [59,60] and for sports-related movements by Skals et al. [61]. Future research is needed to validate the combined use of IMUs and musculoskeletal modeling in a military context, including validation of the energy expenditure, for example, by using indirect calorimetry in aerobic activities. Additionally, the measurement of physiological parameters, such as lactate and heart rate variability (HRV), can help further understand the metabolic and stress load during load carriage [62,63].

Furthermore, other activities and different terrains should be investigated in future studies to get better insights of military performance during a broad range of tasks and situations [64]. The method presented in this study can be directly used for several walking and running activities. However, for some activities (e.g., crawling or climbing over a wall) the method will have to be expanded because, for these activities, the body will not only interact with the floor/environment via the feet but also with other parts of the body (e.g., hands and knees). In those cases, the ground reaction force prediction will not be sufficient.

The possibilities offered by IMUs in combination with musculoskeletal modeling may, in future research, extend to the study of joint loadings. Previous research has pointed out the burden of injuries related to physical loading [1,65], which indicates the importance of further investigation into the effects on the musculoskeletal system. The results can indicate what muscle to train and help in optimizing training schedules to prevent injuries. Additionally, quantification of joint loading and muscle activation is useful for the selection or design of equipment (e.g., exoskeletons) to support military personnel during their activities. Ultimately, the proposed method helps to answer questions related to optimal and efficient load carriage during military deployment and the imposed requirements of the individual soldier.

In conclusion, this study shows that the combined use of IMUs and musculoskeletal modeling provides more insight into the underlying mechanisms and the changes in the musculoskeletal system during physical loading. Contrary to previous research, kinematic parameters could be determined in an ambulatory setting, which makes it possible to use this tool in military practice. The results showed that, with increased loading, completion time and heart rate significantly increase. Additionally, changes in energy expenditure, muscle parameters, and kinematic parameters were found. This study is a first step towards better monitoring of physical performance during the challenging circumstances that military encounter in their work.

## Figures and Tables

**Table 1:** Specifications of the external load segment for the two loaded conditions (M1 and M2).

Measure	M1	M2
Weight on chest (m) (kg)	5.2	15.6
Weight on back (m) (kg)	10.4	15.6
Width (x) (m)	0.31	0.31
Height (y) (m)	0.30	0.30
Thickness on chest (z) (m)	0.0125	0.0375
Thickness on back (z) (m)	0.0250	0.0375

**Table 2:** Completion time and heart rate for ten subjects (*n* = 10), presented as mean ± SD in the case of normal distribution (^†^) or median + range in the case of non-normal distribution, ANOVA test results, and differences between loading conditions. The asterisks indicate significant differences between the conditions based on post hoc test results: * *p* < 0.01, ** *p* < 0.001. Significant results are in bold.

Metric	M0	M1	M2	*p*	M1 rel. M0	M2 rel. M1	M2 rel. M0
CT [s]	11.78 ± 0.46 ^†^	12.32 ± 0.46 ^†^	13.08 ± 0.68 ^†^	**<0.000**	**0.665 (5.6%)** **	**0.700 (5.6%)** *	**1.37 (12%)** **
HR [bpm]	120 + 55	122 + 59	128 + 53	**<0.000**	2 (1.3%)	**7 (5.3%)** *	**8 (6.7%)** *

CT = completion time; HR = heart rate; rel. = relative to.

**Table 3:** Energy expenditure, muscle parameters, and kinematic parameters for three loading conditions of one subject (*n* = 1), presented as median + range, ANOVA test results (significant results are in bold), and the differences between loading conditions.

**Metric**	**M0**	**M1**	**M2**	***p***	**M1 rel. M0**	**M2 rel. M1**	**M2 rel. M0**
**Energy expenditure**							
$P_{met}$ [W kg^−1^]	35.8 + 2.61	39.0 + 0.41	40.0 + 1.78	**=0.050**	3.24 (9.1%)	1.05 (2.7%)	4.29 (12%)
$E_{met}$ [J kg^−1^]	551 + 21.7	589 + 41.8	668 + 5.71	**=0.050**	38.7 (7.0%)	78.3 (13%)	117 (21%)
**Muscle parameters of gluteus maximus muscle**							
$P_{met}$ [W kg^−1^]	0.184 + 0.0747	0.291 + 0.0488	0.319 + 0.0391	>0.050	0.107 (58%)	0.0282 (9.7%)	0.135 (73%)
$E_{met}$ [J kg^−1^]	40.9 + 1.10	48.9 + 1.41	55.8 + 3.44	**=0.050**	8.05 (20%)	6.93 (14%)	15.0 (37%)
$F_{m}$ [N]	124 + 6.90	154 + 7.12	183 + 6.86	**=0.050**	29.7 (24%)	29.3 (19%)	59.0 (48%)
${\dot{L}}_{m}$ [cm s^−1^]	−0.288 + 0.130	−0.770 + 0.294	−0.989 + 0.158	**=0.050**	−0.482 (168%)	−0.219 (28%)	−0.701 (244%)
**Muscle parameters of vastus lateralis muscle**							
$P_{met}$ [W kg^−1^]	0.575 + 0.0416	0.582 + 0.126	0.622 + 0.0399	>0.050	0.0073 (1.3%)	0.0399 (6.9%)	0.0472 (8.2%)
$E_{met}$ [J kg^−1^]	41.8 + 4.03	44.7 + 3.08	48.9 + 0.751	**=0.050**	2.87 (6.9%)	4.22 (9.4%)	7.09 (17%)
$F_{m}$ [N]	316 + 18.9	365 + 71.7	397 + 5.88	**=0.050**	48.9 (16%)	32.0 (8.8%)	81.0 (26%)
${\dot{L}}_{m}$ [cm s^−1^]	0.113 + 0.515	0.127 + 1.13	0.958 + 0.205	>0.050	0.0141 (12%)	0.830 (652%)	0.845 (745%)
**Kinematic parameters**							
$\theta_{trunk}$ [deg]	9.04 + 0.377	8.58 + 1.01	7.79 + 1.45	>0.050	−0.464 (−5.1%)	−0.788 (−9.2%)	−1.25 (−14%)
$ROM_{trunk}$ [deg]	37.9 + 2.82	29.0 + 11.5	28.4 + 2.84	>0.050	−8.82 (−23%)	−0.663 (−2.3%)	−9.48 (−25%)
$y_{CoM}$ [cm]	101 + 1.24	99.2 + 0.66	99.4 + 0.110	>0.050	−1.42 (−1.4%)	0.230 (0.23%)	−1.19 (−1.2%)
$VD_{CoM}$ [cm]	49.1 + 2.40	46.7 + 4.44	44.5 + 1.84	>0.050	−2.42 (−4.9%)	−2.15 (−4.6%)	−4.57 (−9.3%)

$P_{met}$ = metabolic power; $E_{met}$ = metabolic energy; $F_{m}$ = muscle force; ${\dot{L}}_{m}$ = muscle contraction velocity; $\theta_{trunk}$ = trunk angle in the sagittal plane; $RoM_{trunk}$ = trunk range of motion in the sagittal plane; $y_{CoM}$ = vertical position of CoM (center of mass); $VD_{CoM}$ = vertical displacement of CoM; rel. = relative to.

## Data Availability

The data presented in this study are available on request from the corresponding author. The data are not publicly available due to privacy and confidentiality issues.

## Supplementary Materials

The following are available online at https://www.mdpi.com/article/10.3390/s21165588/s1, Video S1: Physical performance analysis by means of inertial motion-capture driven musculoskeletal analysis.
